# Emerging Non-Conventional Approaches in mRNA-LNP Formulation for Therapeutic Applications

**DOI:** 10.3390/pharmaceutics18050527

**Published:** 2026-04-26

**Authors:** Yitian Zhang, Gabriel Linaje-Ferrel, Juan Manuel Rocha Angel, Oindrila Banik, Earu Banoth, Amine A. Kamen, Naresh Yandrapalli, Ayyappasamy Sudalaiyadum Perumal

**Affiliations:** 1 Department of Bioengineering, Faculty of Engineering, McGill University, Montreal, QC H3A 0C3, Canada; yitian.zhang2@mail.mcgill.ca (Y.Z.); hector.linaje-ferrel@mail.mcgill.ca (G.L.-F.); juan.rochaangel@mail.mcgill.ca (J.M.R.A.); 2 OBMS Lab, Department of Biotechnology and Medical Engineering, National Institute of Technology Rourkela, Rourkela 769008, Odisha, India; banikoindrila6@gmail.com (O.B.); banothe@nitrkl.ac.in (E.B.); 3 Viral Vectors and Vaccines Bioprocessing Group, Department of Bioengineering, McGill University, Montreal, QC H2X 1Y4, Canada; amine.kamen@mcgill.ca; 4 Department of Synthetic Biology, Gebaude B2.2, University of Saarland, 66123 Saarbrücken, Germany

**Keywords:** mRNA-LNP, encapsulation, non-conventional methods, microfluidics, hot homogenization, thin film hydration, siRNA, mRNA, machine learning, lyophilization, coacervation, lipid nanoparticles, vaccine development

## Abstract

Lipid nanoparticles (LNPs) have become the cornerstone of nucleic acid delivery platforms, particularly in RNA-based vaccines and therapeutics. However, the conventional methods of LNP production, which are primarily reliant on microfluidic mixing of aqueous and organic solvent phases, pose limitations in terms of mRNA stability, residual organic contamination, scalability, cost, and environmental impact. These limitations prompted a renewed search for non-conventional strategies with the promise of improving mRNA-LNP encapsulation approaches. These emerging approaches aim to address key bottlenecks, including mRNA hydrolysis-driven degradation, high production losses, and complex downstream purification. Moreover, the ability to decouple LNP synthesis from mRNA encapsulation could enable streamlined, modular manufacturing workflows and customizable payload delivery, including single- or multiple-mRNA payloads, thereby expanding the therapeutic scope of LNPs. This review offers an early insight into the design principles and scalability potential of emerging non-conventional LNP encapsulation approaches, including solvent-free and microfluidics-free methodologies, and pre-built LNP workflows. We also examine trends in emerging LNP encapsulation tools, including high-shear mixing, sonication, membrane contraction, and other approaches. Finally, we extrapolate the suitability of the methods for scale-up approaches and their economic implications based on the process information.

## 1. Introduction

Lipid nanoparticles (LNPs) have revolutionized the field of drug delivery, particularly in delivering messenger RNA (mRNA) for vaccine and therapeutic applications [1,2,3,4,5,6,7,8]. The success and unprecedented speed of mRNA-based COVID-19 vaccines further catalyzed a global surge in LNP-related research, driven by their ability to protect labile nucleic acids from hydrolysis and enzymatic degradation, facilitate cellular uptake, and efficiently release them into the cytosol [2,9,10,11]. Despite their success, LNP formulations currently face three areas of substantial technical and economic limitations, namely: (a) materials and sustainability; (b) process-dependent complexities; (c) cumbersome installation and reproducibility issues during scaling and (d) issues with storage and distribution logistics. For example, specific issues include production complexity [12], thermal instability [10,13,14,15], material wastage during scale-up [16,17,18,19], and toxic waste (environmental aspect) [20,21].

**Figure 1:**
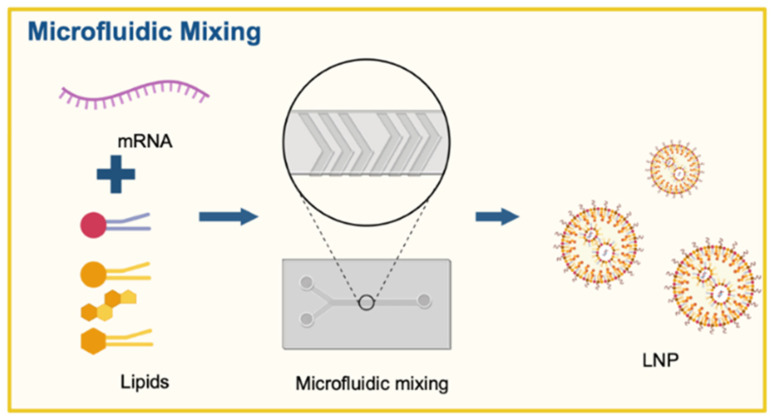
Conventional, solvent-based, microfluidic encapsulation approach. The current microfluidics approach involved two major technologies: Dean mixers [45], a patented workflow from Cytiva (Precision Nanosystems Inc., Vancouver, BC, Canada), and the T-junction-based high-volume, high-speed encapsulation approaches [22,46], involving conventional assembly and setup used in chromatography applications for buffer mixing and liquid handling.

The gold standard for commercial and research-based mRNA-LNP production is the use of microfluidic devices (Figure 1). In this procedure, a lipid dispersion in an organic solvent flows through a channel crossed and pinched by an aqueous solution containing the target mRNA [22,23,24,25,26,27,28]. Even traces of organic solvent residues in encapsulated mRNA-LNP complexes can compromise mRNA integrity, necessitating complex filtration and cleaning steps [14]. Another critical issue is the stability of the encapsulated mRNA, which can undergo degradation through a multi-step process, including oxidation, hydrolysis, and transesterification [29]. These mRNA chemical interactions with encapsulated contents such as lipids, water, and residual organic solvent could cleave the sugar–phosphate backbone of the nucleic acid, rendering it incapable of in vivo translation into a stable protein [29,30]. These damages often occur during the storage of the mRNA-LNPs, as their thermostability remains a significant challenge for maintaining long-term stability [13,14,24,31]. Another contributing factor is the high water content within the core of the ionizable LNPs—i.e., the aqueous phase [27,32,33]. In fact, studies have shown that LNPs may contain up to 20–24% water, which is sufficient to hydrolyze the mRNA [33]. While lyophilization can be used to increase product stability—by reducing residual water within the LNPs—it is a costly and highly energy-demanding process [14,27,30]. Another important way in which mRNA can lose activity is by forming lipid adducts [10,14,34,35,36], i.e., the covalent bonding between reactive lipid species and the nitrogenous base of the nucleic acid [11,29,35]. These bonds modify the secondary structure of the mRNA, leading to a loss of functionality within a cell. Lipid adductions are a primary concern, as most LNP formulations require ionizable lipids, which are highly prone to forming covalent bonds with mRNA and other lipid moieties, making mRNA release difficult [10,14,24,35,37]. Additionally, microfluidic nanoprecipitation generates a significant amount of mRNA waste due to two-fold reasons: (a) need for dead volumes and instrument waste [38,39] and (b) lack of encapsulation efficiency leading to free mRNA or degraded mRNA burdening downstream processing. Microfluidic procedures often pre-set the dead volumes of the aqueous and organic phases to ensure homogeneous mixing. This dead volume typically represents a 15% or greater loss of mRNA for small sample volumes [40]. As mRNA production—at both laboratory and large scales—is costly, any amount of waste can substantially increase production costs [41]. Given these limitations, finding alternative ways to encapsulate mRNA using microfluidics-free, solvent-free approaches is of great importance and has gained renewed interest.

Furthermore, the per-particle encapsulation efficiency of mRNA is low and highly variable, resulting in inconsistent dosages. Of the dosage load, the yield of cytosol-accessible RNA species (both mRNA and siRNA) remains low, often below 2–3%, due to endosomal entrapment and poor escape efficiency [31,42,43]. Additionally, in a large-scale good manufacturing practice (GMP) biomanufacturing facility, the use of highly flammable organic solvents such as ethanol and isopropanol poses significant fire risk hazards [24,44].

**Figure 2:**
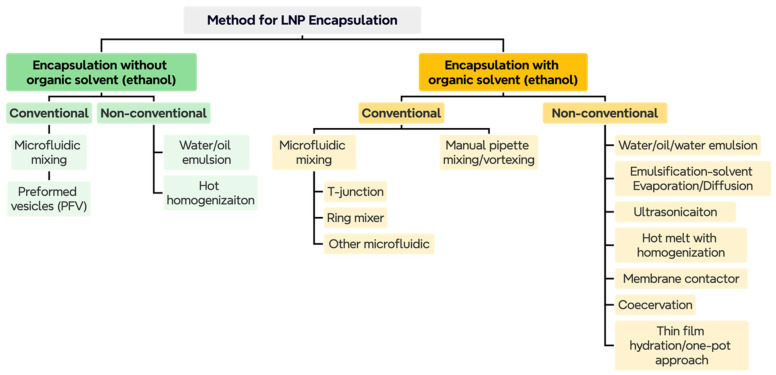
Overview of the conventional and non-conventional methods of LNP synthesis. The flowchart represents the various methods based on the need for solvent and microfluidic devices for the encapsulation approaches. Some of the methods mentioned, like pipette mixing, are still small-scale R&D and academic approaches worth mentioning in the category provided in the flowchart.

To address these limitations, non-conventional approaches have emerged as promising alternatives (Figure 2—overview of different approaches). These approaches encompass both bottom-up and top-down methods, with some utilizing microfluidics-based designs that incorporate additional technological components, such as ultrasonication-in-chip mixing, enhanced design strategies (scale-up or parallel operating concepts), and certain microfluidics-free and solvent-free designs, to address sustainability and safety concerns. Thus, a wide variety of methods are discussed in this paper in the context of RNA therapeutics applications, including spontaneous self-assembly under aqueous conditions, high-energy mechanical methods such as sonication or high-shear mixing, and green chemistry-based processes that eliminate the use of toxic organic solvents. In addition, a new class of modular, prebuilt vesicles or prefabricated LNPs (PFVs) enables decoupled post-synthesis loading of siRNA (predominantly), mRNA, or other nucleic acids. This two-step formulation strategy significantly improves mRNA integrity by minimizing exposure to reactive lipid intermediates and organic solvents, thereby enhancing shelf-life and delivery efficiency [30,47,48]. These strategies are technically innovative and could be economically favourable. Solvent-free techniques reduce the need for resource-intensive downstream purification and solvent recovery, while PFVs lower production waste and enable flexible, on-demand cargo loading. Importantly, these platforms are adaptable for both single-payload systems, optimized for focused therapeutic applications, and multi-component and multi-payload delivery, which is essential for complex diseases requiring synergistic gene expression or the co-delivery of mRNA targets for developing multivalent vaccines [49].

As the field progresses, the convergence of solvent-free synthesis, modular LNP assembly, and advanced analytics promises to reshape the landscape of RNA therapeutics. This review aims to comprehensively evaluate these emerging LNP strategies, with a specific focus on the process conditions, synthesis approaches, biochemical underpinnings, economic feasibility, and translational potential to large-scale production systems. The rationale for a review of this specific topic is that non-conventional, emerging approaches are significantly expanding, compared with mainstream LNP encapsulation using microfluidic mixing technologies. Thus, this review explains various non-conventional approaches, their impacts, and the scalability from lab scale to commercial scale, as well as their operational processes. Apart from the texts explaining recent progress in mRNA, the work also provided comprehensive tables that summarize key information on these emerging techniques, including scale-up feasibility based on reported research and review work.

## 2. Overview of LNP Synthesis Methods

Figure 2 provides a comprehensive overview of the current landscape of LNP synthesis methods, categorizing them into two broad groups: those that use organic solvents, such as ethanol, to solubilize lipids and other ingredients, and those that are organic solvent- or solvent-free. Both categories encompass both conventional and non-conventional approaches, which are further divided into numerous subcategories and types. Conventional LNP synthesis techniques rely on ethanol as a solvent and on microfluidic mixing [27,50], which are highly effective for encapsulating biomolecules such as drugs or nucleic acids. Rapid mixing through microfluidics is a bottom-up, conventional manufacturing process. However, there is an increasing demand for more environmentally sustainable alternative methodologies [51,52].

Several works have focused on conventional microfluidic mixing approaches, which are widely adopted [22,25]. But recent advancements have introduced a diverse array of non-conventional methods aimed at overcoming key limitations as described above (in the Introduction Section), including solvent-associated toxicity, low encapsulation efficiency, and poor scalability, as well as techno-economic constraints. This review emphasizes the emerging non-conventional methods as the field rapidly expands to incorporate complex mRNA therapeutic molecules. Figure 2 illustrates the comparative breadth of conventional versus non-conventional LNP synthesis strategies. While traditionally applied to small molecules and hydrophobic drugs, many of these methods are now being re-engineered for large biomolecules such as mRNA and CRISPR-Cas co-encapsulation components. Notably, several microfluidics-free systems, such as hot-melt extrusion coupled with high-pressure homogenization, flash nanocomplexation, and membrane contactor methods, are now being optimized to overcome the challenges posed by ethanol dependency while preserving the favourable nanoparticle attributes. These adaptations reflect a broader trend towards modular, scalable, and environmentally conscious manufacturing pipelines for next-generation nucleic acid therapeutics. Further, this review primarily examines the relevance of emerging non-conventional approaches to mRNA encapsulation in LNPs. However, in certain sections it was necessary to include pioneering studies using closely related nucleic acid cargos (e.g., siRNA or plasmid DNA) and, in rare cases, hydrophobic small-molecule payloads. These studies are cited to provide historical and mechanistic context and to clarify how specific strategies may be extendable to mRNA encapsulation, while explicitly noting when direct mRNA validation is not yet available.

Taken together, Figure 2 not only maps the diversity of existing LNP synthesis strategies but also highlights a clear evolutionary trend, from solvent-dependent, microfluidics-based systems toward greener, solvent-free innovations. This shift reflects a broader rethinking of how lipid self-assembly, encapsulation, and scalability can be achieved with fewer environmental and operational constraints.

## 3. Non-Conventional Emerging Methods and Their Impacts as Emerging Approaches for LNP Formulation

While microfluidic and ethanol injection methods currently dominate mRNA–LNP manufacturing, comprehensive reviews have detailed these conventional approaches, along with their critical process parameters (Table 1), downstream purification strategies, and Process Analytical Technology (PAT) frameworks [53,54,55,56]. Readers seeking in-depth analyses of such microfluidics-based techniques and scale-up strategies may refer to highly cited sources discussing microfluidic mixing geometries, T-junction scaling, and orthogonal PAT tools such as Dynamic Light Scattering (DLS), Nanoparticle Tracking Analysis (NTA), and Analytical Ultra-Centrifugation (AUC) [19,22,23,24,25]. In contrast, the following sections focus exclusively on emerging non-conventional solvent-free and microfluidics-free approaches for mRNA-LNP encapsulation, including case studies and reported outcomes from such mRNA delivery studies. Each method is described in the context of background information on cargoes (some mRNA-relevant cargoes, such as siRNA and similar charge-character cargoes, like plasmid DNA), formulation strategy, process conditions, encapsulation performance analytics, and translational potential, with a focus on feasibility for scale-up, particularly for mRNA encapsulation.

### 3.1. Microfluidics-Free Manufacturing Methods

**Figure 3:**
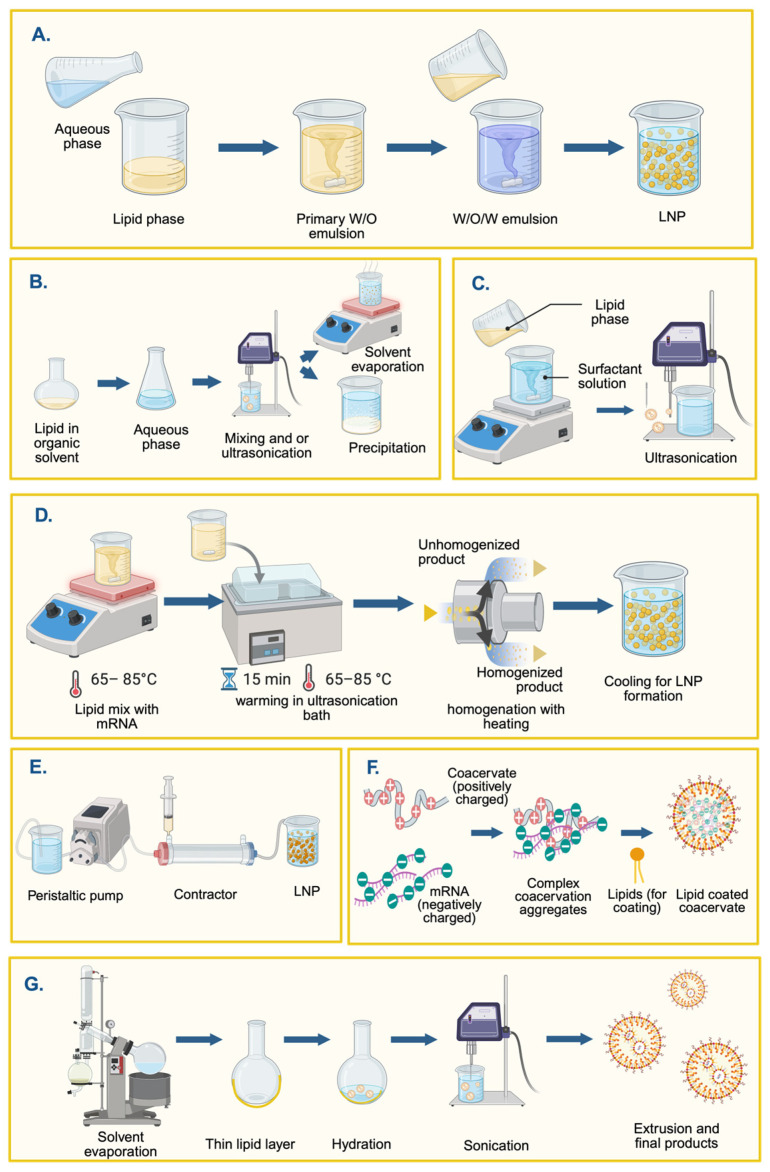
Different methods of non-conventional LNP-mRNA encapsulation approaches. The figure schematically presents 7 different non-conventional, non-microfluidic approaches that are less explored, with some of them as emerging methods for LNP encapsulation. (**A**). Water/oil/water emulsion; (**B**). Emulsification-solvent evaporation/diffusion; (**C**). Ultrasonication; (**D**). Hot melt with homogenation; (**E**). Membrane contractor; (**F**). Coacervation; (**G**). Thin film hydration/one-pot approach.

Microfluidics-free LNP synthesis can be categorized into two sections: top-down methods, such as extrusion, high-pressure homogenization (HPH) [50], and sonication [54], and bottom-up methods, including thin-film hydration and supercritical fluid-based methods (Figure 3) [53]. Top-down approaches are generally high-energy methods that rely on generating high shear forces by subjecting lipids to harsh conditions, such as high pressure or high temperature, to reduce LNP size. The bottom-up approach starts with the foundational components of LNPs, such as lipids or polymers, and further self-assembles them into larger LNPs. Some of the other bottom-up approaches include thin-film hydration, the Sprayed Multi-Absorbed-droplet Reposing Technology (SMART) intrusion-based approach [55], and solvent-free self-assembly, including coacervation, which are further detailed below.

#### 3.1.1. Emulsions and Nanoemulsions

Nanoemulsions are submicron emulsified systems (<200 nm) that have gained attention for nucleic acid delivery due to their tunable size, thermodynamic stability, and potential to encapsulate both hydrophilic and lipophilic payloads. These systems can be classified into three primary configurations: (a) simple emulsions (oil-in-water, O/W or water-in-oil, W/O), (b) double emulsions (water-in-oil-in-water, W/O/W), and (c) multicore or multiple emulsions, each offering distinct advantages for mRNA or DNA encapsulation, protection, and release kinetics [57]. W/O emulsions, particularly in their double-emulsion (W/O/W) format, remain a foundational method for encapsulating hydrophilic, negatively charged payloads, such as mRNA, nanoparticles [57] and other complex biomolecules [58]. This technique involves emulsifying (primarily through high-speed stirring or mixing) an aqueous drug solution within a molten lipid phase, often stabilized by surfactants (Figure 3A). Particularly for hydrophilic payloads, such as mRNA, a double emulsion (W/O/W) strategy can be employed to encapsulate the payload within an aqueous core surrounded by lipid shells. This configuration supports sustained release and payload protection, although it is suggested to be susceptible to instability (Ostwald ripening and coalescence) and challenging to scale [57]. For acid (mRNA) delivery, these emulsions have been explored in exploratory formulations, employing a W/O/W approach to preserve mRNA integrity in models. However, robust and scalable mRNA studies remain necessary to confirm the method’s utility [59,60]. Several studies have recently explored the emulsion approach, using various renewable and non-renewable raw materials for mRNA encapsulation [57,58,61].

Mok et al. developed a PEG-assisted single O/W emulsion method to encapsulate plasmid DNA in PLGA nanospheres by first forming PEG/DNA nanocomplexes that are soluble in an organic solvent (80% methylene chloride/20% DMSO). The resulting <100 nm complexes produced a clear, aggregate-free organic phase and improved DNA loading, particle sizes and nanosphere structural integrity [61]. Adopting this approach, the effectiveness of encapsulating mRNA was tested by Xu et al. (2024) [62], who demonstrated that mRNA-PLGA nanoparticles coated with activated dendritic cell (DC) membranes enhanced antigen delivery and immune response duration. The core nanoparticles were synthesized via a modified single emulsion–solvent evaporation method, encapsulating ovalbumin (OVA) as a model antigen. The resulting mRNA-PLGA nanoparticles exhibited an average size of ~128.3 nm, a polydispersity index (PDI) of 0.076, and a zeta potential of −26.5 mV, indicating monodispersity and a surface charge suitable for colloidal stability. After membrane coating, the particle size slightly increased to 138.8 nm while maintaining a low PDI (0.082), confirming successful membrane fusion without aggregation and an encapsulation efficiency of approximately 85.6% [62]. A study by Borrajo et al. (2024) [58] investigated nanoemulsions (NEs) and nanocapsules (NCs) as alternatives to lipid nanoparticles for the delivery of intranasal mRNA vaccines. This study developed and screened a diverse library of nanocarriers for mRNA protection and transfection efficiency. Two promising formulations were identified: an NE formulation composed of C12-200, DOTAP, and DOPE, with a particle size of 120 nm and a surface charge of +50 mV, and an NC formulation using the same lipid components with a dextran sulphate coating, achieving a size of 130 nm and a surface charge of −16 mV. Upon intranasal administration of mRNA encoding for ovalbumin, both systems induced strong CD8+ T cell responses, demonstrating their potential for needle-free mucosal mRNA vaccine platforms. This study utilized lipids and mRNA formulated in a blended organic–aqueous emulsion, employing bulk mixing, i.e., high-shear mixing [58].

Similar to the W/O/W emulsion shown in Figure 3A, the emulsification–solvent evaporation/diffusion approach (Figure 3B) is also used in LNP synthesis. The lipid components are dissolved in organic solvents such as ethanol, and the lipid phase is then mixed with the aqueous phase containing mRNA or siRNA, and the mixture is homogenized using a mixer. To extract the LNP, the mixture can be either evaporated by heating or precipitated under air-dry conditions.

#### 3.1.2. Hot Melt Approaches with High-Pressure Approaches

Hot homogenization involves dispersing a drug into melted lipids and emulsifying at elevated temperatures before forcing the mixture through high-pressure homogenizers or rapid mixing (such as pipette mixing) to produce nanosized droplets (Figure 3D). Advantages include solvent-free conditions and scalability, making it suitable for lipophilic drugs. Disadvantages include exposing sensitive molecules—such as nucleic acids—to heat, which may degrade them or alter the crystalline phase of the lipid. While commonly used for small-molecule delivery, for instance, Stavudine–LNP scaling [63,64], its application to mRNA is limited by the heat sensitivity of nucleic acids. It requires further optimization, such as temperature control or the use of RNA stabilization agents, to expand into mRNA delivery.

This hybrid technique fuses continuous hot-melt extrusion of lipid–payload blends with immediate size reduction via high-pressure homogenization. It offers a single-line, solvent-free, scalable process—but is generally restricted to thermostable actives. Hot melt extrusion with hot homogenization (HME) parameters, such as extrusion temperature, screw design, and residence time, significantly influence the nanoparticle’s size and encapsulation efficiency [65] because these factors contribute to process variation and deviations. This high-energy technique in LNP production involves melting lipids above their transition temperature (typically 5–10 °C above) and forming a hot O/W pre-emulsion with the payload and surfactants. This pre-emulsion is then forced through a narrow gap under high pressure (100–2000 bar), generating shear and cavitation forces that reduce droplets to the nanometre scale; subsequent cooling solidifies the particles [66,67]. Originating from applications in solid lipid nanoparticles (SLNs) and nanostructured lipid carriers (NLCs), hot-high-pressure homogenization has demonstrably improved encapsulation efficiency for hydrophilic drugs—for instance, Kasongo et al. (2012) reported a rise in encapsulation efficiency of didanosine-loaded NLCs from 32% to approximately 52%, with a concomitant increase in loading capacity, by sequentially using hot and cold homogenization steps, underscoring the method’s adaptability and effectiveness in drug encapsulation contexts [68].

Building on these reports towards mRNA encapsulation, emerging literature explores adapting hot homogenization for mRNA encapsulation within LNPs. Although direct mRNA studies remain limited, Yun et al. (2025) highlighted recent advances in functional lipid-based nanomedicines, recognizing the potential of hot homogenization variants to process thermostable lipid–RNA complexes and facilitate organ-specific delivery through controlled thermal and mechanical inputs [69]. Wang et al. (2024) emphasized that careful lyophilization after high-temperature LNP processing is necessary to maintain particle integrity, size uniformity, and mRNA stability post-encapsulation [70]. Furthermore, Goli et al. demonstrated the use of nanostructured lipid carriers (NLCs) for co-encapsulation of artemether (ARM) and miRNA, showing a promising hybrid strategy for drug and nucleic acid delivery. Their process used dynamic high-pressure homogenization with pre-emulsion lipid melts, yielding stable LNPs with an average size of ~94.4 nm (ARM + miRNA), a PDI of ~0.12, and a zeta potential of −11.8 mV. Remarkably, they reported an encapsulation efficiency of 93.06 ± 3.43%, demonstrating that such thermal and shear-driven methods can effectively retain and stabilize sensitive nucleic acids within lipid matrices. While their study focused on drug plus miRNA, the results parallel those of mRNA’s encapsulation dynamics, highlighting the possibility of co-loading strategies in infectious and neuroinflammatory diseases [71]. Patel et al. (2026) corroborated these directions in an industrial context, discussing the integration of hot-melt extrusion with homogenization for continuous, scalable mRNA vaccine production [72]. Together, these findings position hot homogenization not only as a key method for small-molecule and protein delivery but also as an adaptable, energy-efficient, and scalable platform for mRNA-LNP production—provided lipid phase transitions, nucleic acid compatibility, and process conditions are rigorously optimized. For example, Krzysztón et al. demonstrated that siRNA-loaded LNPs produced using a hydrodynamic flow focusing (HFF) device achieved a 20% increase in encapsulation efficiency for 38 nm particles compared with bulk mixing methods. However, in HFF systems, such small particles are typically obtained only at Q ratios > 30, which results in significant dilution of the final formulation [73]. To date, no studies have successfully encapsulated mRNA using hot homogenization or hot-melt coupled high-pressure techniques for mRNA-LNP production.

#### 3.1.3. Membrane Contactor

Membrane contactors represent an advanced top-down, scalable engineering approach for producing LNPs with controlled size and scalability. In this technique, the molten lipid phase is pressed through the pores of a specialized porous membrane, typically at temperatures above the lipid’s melting point. In contrast, an aqueous phase flows tangentially across the membrane surface (Figure 3E). The aqueous stream sweeps away nascent lipid droplets that form at the membrane outlet, which then solidify upon cooling to create solid lipid nanoparticles. In the foundational study by Charcosset et al., operating parameters, including lipid and aqueous phase temperatures, transmembrane pressure, crossflow velocity, and membrane pore size, were systematically varied. This allowed for the precise tuning of particle sizes (ranging from approximately 70 to 215 nm) and lipid flux (0.15–0.35 m^3^/h·m^2^) while ensuring ease of operation and feasibility for scale-up [74].

In a recent study, Atallah et al. extended this technology to the synthesis of mRNA-loaded LNPs using DOTAP as the cationic lipid model. Their membrane micromixer, operating at flow rates of up to 1000 mL/min, significantly higher than those of typical microfluidic systems, achieved remarkably consistent particle sizes in the 103–118 nm range, with PDI values consistently below 0.2. This high-throughput nanoextrusion was performed using a 10 µm pore SPG membrane, and flow rates as low as 500 mL/min were sufficient to produce LNPs of suitable quality [75]. Ethanol-to-aqueous phase volume ratios (Veth/Vaq) of 1/4 and 1/6 were tested to examine solvent reduction impacts. Despite the higher lipid concentration required at lower ethanol content (14 mg/mL at 1/6 vs. 10 mg/mL at 1/4), both ratios yielded particles with comparable size (≈115–120 nm) and narrow PDIs (≈0.20), indicating that the micromixing zone within the annular membrane geometry enabled consistent nucleation even under supersaturated conditions. Post-treatment processing, including sterile filtration, further reduced the size to 103 ± 5 nm without affecting encapsulation efficiency (EE), which remained exceptionally high (~96%). While processing steps, such as dialysis, led to mRNA yield losses due to degradation, the membrane micro-mixing approach successfully produced stable mRNA-LNP formulations with minimal structural defects (e.g., blebs). It sustained physical stability over 2 months at 4 °C. This demonstrates membrane micro-mixing as a compelling candidate for continuous, GMP-compatible manufacturing of mRNA-LNPs, extrapolated based on the recent successful demonstration.

#### 3.1.4. Coacervation

Coacervation is a phase-separation technique in which a solution separates into a dense, macromolecule-rich “coacervate” phase and a dilute phase. This phase enrichment enables increased molecular density, a crucial feature for achieving higher dosages per particle. Traditional LNP production methodologies struggle to encapsulate a large number of mRNA molecules per particle [76]. However, complex coacervation involves condensation of mRNA [77,78]. Complex coacervation involves two or more oppositely charged polymers (or polyelectrolytes) that electrostatically attract each other and separate into a polymer-rich liquid phase [79]. The driving force behind complex coacervation is the release of counterions, which increases entropy as oppositely charged polymers condense. The mRNA, being a highly charged molecule, can be condensed in the presence of oppositely charged species such as a positively charged (bio)polymer. Multiple researchers have shown that such coacervation can achieve biomedical applications, such as inflammation treatment, wound healing, and even cancer treatment [80]. However, coacervation has a major drawback: it is sensitive to pH, salt, and colloidal stability [81]. While LNPs exhibit greater resistance to the issues above, they lack higher encapsulation efficiency; coacervation exhibits greater encapsulation efficiency but poorer stability. Thus, researchers have combined the two technologies to develop a hybrid LNP production methodology that does not require harsh organic solvents or microfluidic devices, but rather simple mixing to form dense mRNA coacervates, followed by lipid coating. From Figure 3F, the process involves electrostatic self-assembly, in which a negatively charged nucleic acid (such as mRNA) is mixed with a positively charged polymer or polycationic peptide [77]. Upon mixing, oppositely charged macromolecules spontaneously condense into a coacervate-rich nano- or microdroplet phase. The mRNA, being polyanionic, becomes encapsulated and protected within the dense coacervate core by the electrostatic binding to the polycation. In the next step, the prefabricated liposomes are allowed to fuse or assemble on the coacervate surface [82,83]. Due to the simplicity of the methodology, particles can be produced using multi-step high-shear homogenization and other mixing techniques, thereby eliminating the need for specialized microfluidics.

#### 3.1.5. One-Pot Solvent-Free Synthesis and Thin Film Hydration

Both one-pot solvent-free synthesis and thin film hydration (TFH) represent solvent-minimized strategies for LNP formation. Yet, they differ fundamentally in mechanism, process design, scalability, and encapsulation behaviour, despite relying on self-assembly. In the TFH method (Figure 3G), lipids are first dissolved in an organic solvent, such as chloroform or ethanol, and subsequently evaporated under vacuum to form a uniform, dry lipid film on the flask surface [84]. Upon hydration with an aqueous buffer, the amphiphilic lipids spontaneously reorganize into multilamellar vesicles (MLVs). These are then downsized using sonication or extrusion to produce unilamellar liposomes or nanoparticles. The process is largely mechanical and sequential, involving distinct stages of solvent removal, hydration, and size reduction.

By contrast, the one-pot solvent-free approach eliminates the solvent and film formation stages. Instead, all components—lipids, nucleic acids, and buffers—are combined simultaneously in a single aqueous vessel [72]. Under mild heating (typically 37–45 °C) and gentle stirring, spontaneous self-assembly occurs. Electrostatic interactions between ionizable cationic lipids and negatively charged nucleic acids drive the formation of bilayer or core–shell LNP structures. The process is thermodynamically driven and continuous, relying on chemical self-organization rather than mechanical disruption. The TFH method relies heavily on organic solvents to dissolve and deposit lipids, introducing environmental and safety concerns and requiring solvent recovery or disposal. In contrast, the one-pot method is entirely aqueous, aligning with the principles of green chemistry [85]. The absence of organic solvents not only reduces environmental burden but also simplifies purification, as no solvent removal or dialysis is required. LNPs produced by TFH often exhibit heterogeneous size distributions and low encapsulation efficiency for hydrophilic or large payloads such as mRNA, because encapsulation occurs only after vesicle formation. Additional processing, such as extrusion or freeze–thaw cycling, is often required to achieve desired particle sizes (50–150 nm). In the one-pot approach, however, nucleic acid encapsulation occurs concurrently with vesicle formation, resulting in tighter lipid–RNA complexes and potentially higher encapsulation efficiency, especially for small oligonucleotides, such as siRNA. Nevertheless, because the assembly kinetics are diffusion-controlled, particle uniformity and reproducibility remain challenging without precise control of parameters such as ionic strength and lipid ratio [85].

### 3.2. Microfluidics-Based Non-Conventional Manufacturing Methods

**Figure 4:**
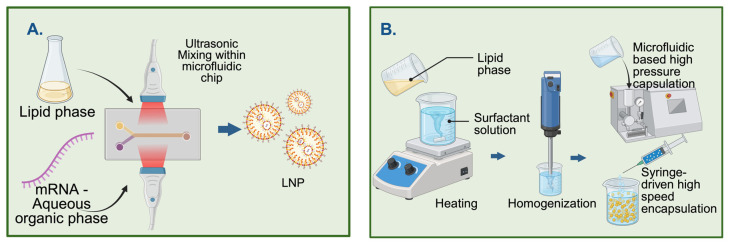
Different methods of emerging microfluidics-based LNP-mRNA encapsulation approaches. Schematically illustrates non-conventional, microfluidics-dependent, yet approaches for LNP encapsulation. The approach proposed here essentially involves value addition to the existing setup to achieve efficient RNA-type encapsulation. (**A**). Microfluidic mixing with on-chip ultrasonication; (**B**). High pressure homogenization (cold). Adopted and reworked from [63].

**Figure 5:**
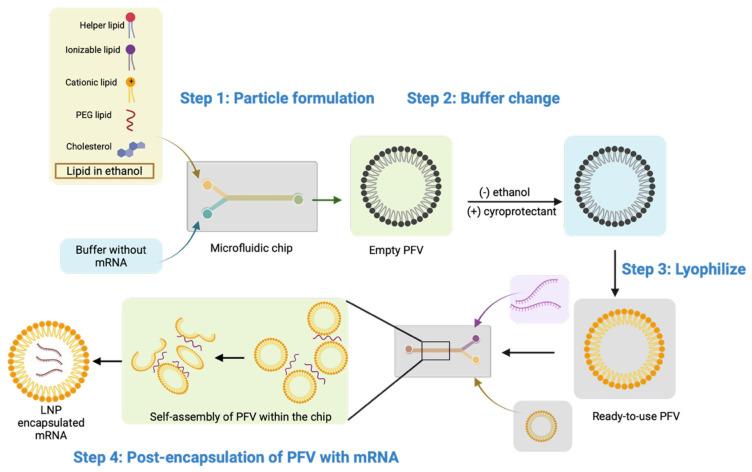
Prefabricated LNP vesicles. Workflow of PFV production and post-encapsulation of PFV with mRNA. PFV-mRNA LNP represents a recent, modular approach for encapsulation using prefabricated lipid vesicles. Currently established for siRNA, its practicality remains to be tested for mRNA.

This section describes non-conventional microfluidics-based approaches that typically involve a value-added microfluidic device strategy to enhance encapsulation ratios, fine-tune sizes, modularity, or consider sustainability from a broader perspective (Figure 4 and Figure 5). This section is described into three major categories, as follows.

#### 3.2.1. Microfluidic Ultrasonication

Previous literature has shown that the immune response to the administered mRNA-LNPs is influenced by particle size [86]. In fact, 160 nm nanoparticles have been shown to elicit greater immune rejection than similar particles of smaller size (20 and 90 nm) [86]. Current standard microfluidic devices are challenged by clogging and limited throughput [87]. Furthermore, while microfluidic ultrasound-assisted synthesis (Figure 4A) has been attempted before, many of these systems suffer from inhomogeneous cavitation [87]. Therefore, the development of a high-throughput system capable of producing homogeneously sized particles below 100 nm is required. To address these limitations, Liu et al. developed a microfluidic ultrasonic cavitation (MUC) device [87]. Leveraging precise mixing in laminar-flow microchannels and ultrasonic energy to refine particle size, this method ensures the consistent production of homogeneous LNPs with narrow polydispersity and high encapsulation efficiency. While microfluidics has already transformed mRNA vaccine production (e.g., COVID-19 LNPs), thanks to precise and repeatable mixing [24,27,28], the addition of an ultrasound step provides further refinement. Although throughput remains limited without chip multiplexing, this method is highly relevant for mRNA applications, where batch consistency is crucial for accurate results. Conceptual images of the process are provided in Figure 3C and Figure 4A for the one-pot and microfluidic approaches, respectively.

#### 3.2.2. High-Pressure Syringe-Driven Approach

The SMART (Syringe-based Microfluidics-Assisted Rapid Technology) combined with the MaGIC (Microfluidics-assisted Geometry-Induced Complexation) platform represents an emerging and unconventional approach to fabricating LNPs for mRNA delivery [55]. This technique (Figure 4B) uses manual syringe extrusion through a narrow 21G (⌀ 0.4 mm) nozzle under relatively high pressure (~200 kPa), resulting in fine mixing conditions for the formation of a complex between lipid and mRNA components. Lipids composed of DSPC:Chol:DOTAP:DMG-PEG (10:48:40:2) were dissolved in ethanol (6 mg/mL) and extruded into an aqueous mRNA solution (10 mM citrate buffer, pH 6.0) at a 2:1 volume ratio, yielding a final lipid concentration of 2 mg/mL. The mixture was extruded a second time to ensure complete encapsulation before dialysis (MWCO 3.5 kDa) to remove ethanol. Notably, integration of the MaGIC nozzle downstream enhanced complexation efficiency and controlled particle size by inducing additional hydrodynamic focusing and shear.

The particle formulations were optimized by varying the amine group from an ionizable lipid to a phosphate group from the cargo (N/P) ratio, ranging from 0.1 to 1.25, enabling fine-tuning of electrostatic interactions between cationic lipids (e.g., DOTAP) and the negatively charged mRNA. This modular syringe-based process enables parallel and benchtop-scale preparation with a low equipment footprint, thereby enabling rapid prototyping of multiple formulations without the need for microfluidic chips or large-scale homogenizers [55]. From a performance standpoint, the SMART-MaGIC approach allows precise size control (via nozzle geometry and pressure), relatively low sample volumes, and easy adaptation of lipid composition. The process accommodates biologically relevant lipid ratios and is compatible with PEGylated and cationic lipid systems, making it suitable for mRNA vaccines. However, there are notable limitations: the extrusion pressure (~200 kPa) is lower than that of industrial high-pressure homogenization but higher than that of gravity-fed or passive mixing, potentially requiring reinforcement of syringe components or precision extrusion systems for scale-up. Furthermore, reproducibility across batches could be challenging without automated actuation or real-time pressure regulation. The reliance on manual syringe control limits throughput and standardization, making this method more suitable for preclinical formulation screening than GMP-compliant manufacturing.

In summary, the SMART-MaGIC technique offers a hybrid of pressure-driven and geometry-modulated complexation, filling a niche between benchtop nanoprecipitation and high-throughput microfluidics [55]. It presents a promising platform for optimizing mRNA-LNP formulations, where rapid prototyping, component versatility, and formulation flexibility are key, particularly in early-stage vaccine or gene therapy development pipelines.

#### 3.2.3. Prebuilt LNPs for mRNA Encapsulation

Post-encapsulation refers to the process of loading preformed vesicles (PFVs)—in this case, LNPs—with the target nucleic acid in a two-step process (Figure 5), as opposed to the current one-step process gold standard, such as in microfluidics (Figure 1) [30]. This sequential procedure, first proposed by J. A. Kulkarni et al., addresses some of the limitations of microfluidics [88]. The mRNA has reduced exposure to organic solvents during manufacturing and to lipids during storage [30]. This methodology has already been integrated into the literature; for instance, J. Duffrène et al. compared conventionally prepared mRNA-LNPs with post-encapsulated mRNA-LNPs for both a droplet injection method and a commercialized complex system [30]. The conventional mRNA-LNPs were then prepared by flowing the lipid infusion (SM-102:DSPC:Chol: DMG-PEG2000 (50:10:38.5:1.5 mol%) in two of three inlets and the mRNA solution (25–100 µg/mL) in the remaining inlet. The aqueous and organic phases were then mixed at a total flow rate (TFR) of 2 mL/min and a flow rate ratio (FRR) of three, followed by dialysis and ultra-centrifugation. The formation of the PFVs followed the same procedure described, but without the mRNA in the aqueous phase; instead, dialysis was performed against an acidic solution, rather than a neutral one. The post-encapsulation of mRNA was performed by diluting the mRNA in either sodium acetate or sodium citrate buffer. Then, the mRNA was encapsulated either by a microfluidic method—droplet-injection chip with a TFR of 2 mL/min and an FRR of one—or by a manual pipetting method, where mRNA was added dropwise into the PFV solution at a 1:3 *v*/*v* ratio under vigorous vortexing. The commercial preparation used the same reagents, but the assembly was now performed using the Nano-Assembler Ignite and the NxGEn microfluidic cartridges; post-encapsulation followed the method described above. Characterization of the particles showed no significant difference between the conventionally made and PFV-based mRNA-LNPs in terms of the physicochemical characteristics of the particles, as well as the in vitro expression and in vivo behaviour [30]. These results highlight the potential of PFVs as an improvement over conventionally synthesized mRNA-LNPs. The group further studied the viability of a delayed post-encapsulation into PFVs. They were able to demonstrate that the PFVs could be stored for at least a month—and potentially for 6 months—and remain viable after encapsulation. In another study, H. Tanaka et al. proposed Ready-to-Use empty freeze-dried LNPs (LNP (RtoU)) to which mRNA can be later encapsulated [47,48].

The proposed four-step process is illustrated in Figure 5. In this method, manufacturers will be responsible for the first three steps, which include the synthesis, buffer exchange, and freeze-drying of the LNPs. The final step involves rehydrating the LNP solution for mRNA encapsulation. The group demonstrated that, for a 96-well format, 25 ng of mRNA was sufficient for successful transfection in HeLa cells and BALB/c mice [47]. While the formulation of LNPs is inflexible, making it unsuitable for applications requiring very specific formulations, the use of LNPs (RtoU) provides an efficient platform for proof-of-concept production [47,48]. This same group later improved on this method by changing their freeze-dried LNPs (RtoU) to liquid-type LNPs (RtoU) (LNPs (RtoU/Liq)). This change eliminates the lyophilization step required for freeze-dried LNPs (RtoU), which could damage the mRNA [47]. For LNPs (RtoU/Liq), the user can mix aqueous solutions of the PFVs and the mRNA [47,48]. The group discovered that the post-encapsulation process is highly pH-sensitive, with a pH requirement of 6.0 for successful encapsulation [47]. The group demonstrated that commercial luciferase-mRNA could be encapsulated with more than 70% encapsulation efficiency using three different ionizable lipid formulations and showed comparable transfection efficiency in vivo and in vitro when compared to conventional mRNA-LNPs [47]. Another promising result for a PFV came from Y. Eygeris et al. [49]. In this study, PFV-LNPs were used to deliver retinal mRNA [49]. The team employed a similar microfluidic approach to both create the PFVs and post-encapsulate, as described by J. Duffrène et al. [30]. While the work showed that PFVs had a lower encapsulation efficiency (around 50%), the approach still demonstrated improved EGFP transfection in mice when administered via subretinal injection. These PFVs also showed potential as delivery systems for gene editing, exhibiting greater cell tolerability and transfection efficiency in mice than conventional LNPs. Interestingly, post-encapsulation of mRNA resulted in a new blebbed morphology, hypothesized to be due to the additional cargo and the charge-induced LNP restructuring and dynamic modifications at the structural level.

### 3.3. Self-Assembly and Green Chemistry-Based LNP Production

The green chemistry approaches to LNP encapsulation take two distinct viewpoints: (a) the use of lipid assembly processes that reduce dependency on organic solvents; (b) the use of renewable materials for LNP-mRNA encapsulation is advantageous, as the current approach relies on expensive components such as lipids and cholesterol. This section provides a detailed overview of the first approach, recent developments, and sustainable encapsulation approaches. The key development of lipid nanoparticle (LNP) synthesis marks a transition toward spontaneous self-assembly processes, which avoid the use of organic solvents such as ethanol, representing a significant step in green chemistry and sustainable nanomedicine manufacturing. Traditionally, LNP formation relies on the rapid mixing of lipid–ethanol solutions with aqueous nucleic acid solutions, where the polarity shift triggers lipid aggregation and encapsulation of nucleic acids. However, this process requires large volumes of ethanol, necessitating extensive purification and solvent recovery steps that increase cost and environmental impact. In contrast, Kulkarni et al. introduced a solvent-free self-assembly mechanism that fundamentally redefines how LNPs form and encapsulate RNA [88]. This two-stage process utilizes electrostatic interactions and membrane reorganization, rather than solvent-driven nucleation, as illustrated in Figure 1, and further described below.

In the first stage, lipids with a defined molar ratio of 50/10/38.5/1.5 (KC2/DSPC/Cholesterol/PEG-lipid) are hydrated in a pH 4 acetate buffer using a T-junction mixer. The ionizable cationic lipid KC2 becomes protonated at this low pH, imparting a strong positive surface charge to the nascent lipid vesicles. The mixture is subsequently dialyzed against neutral pH (7.4) phosphate-buffered saline (PBS), which stabilizes vesicular structure while maintaining a residual surface charge. The resulting vesicles, named preformed vesicles (PFVs), serve as modular lipid assemblies that can interact with nucleic acids in a controlled second step. At this point, PFVs exist as stable, positively charged unilamellar vesicles. The inclusion of PEG-lipid prevents uncontrolled aggregation and maintains colloidal stability by introducing steric repulsion between vesicles.

**Figure 6:**
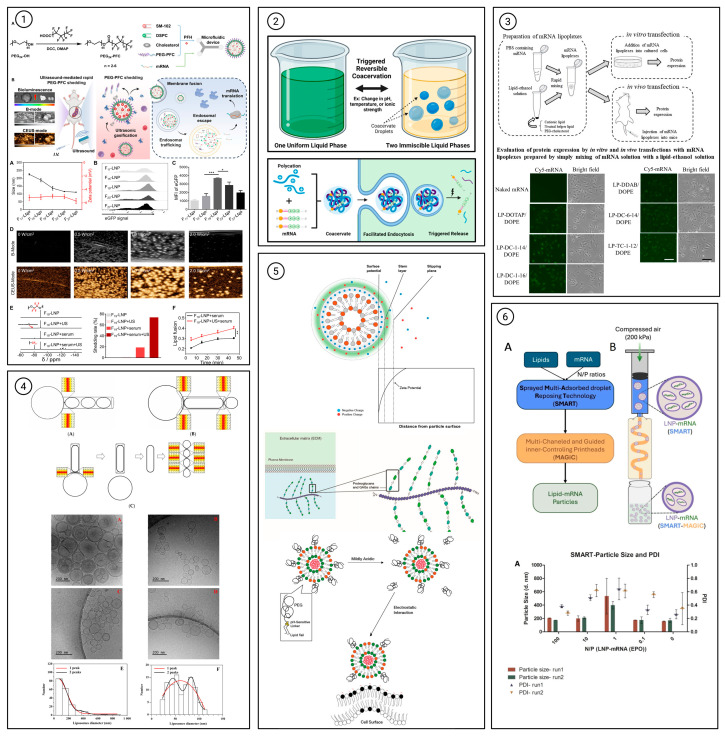
Non-conventional emerging approaches for mRNA-LNP encapsulation and the relevant analytics. The figures (sub-figures with numbers representing different studies) illustrate recent trends in using non-conventional approaches for LNP-based encapsulation, accompanied by respective analytics, including LNP size and in vitro testing for encapsulation efficiency: ①. Ultrasonically cavitating-based encapsulation (* *p* < 0.05, ** *p* < 0.01, *** *p* < 0.001) [90]. ②. pDNA and mRNA-coencapsulation by coacervation [91]. ③. Lipoplex encapsulation of mRNA [92]. ④. mRNA encapsulation by membrane extrusion [93]. ⑤. Electrostatic charge-based encapsulation [94]. ⑥. SMART-injection-based intrusion mRNA-LNP encapsulation [55]. The sub-figures are reproduced with permission from [55,90,91,92,93,94].

In the second stage, siRNA in a pH 4 buffer is introduced into a second T-junction mixer to interact with the PFV suspension. The negatively charged phosphate backbone of the siRNA electrostatically binds to the positively charged KC2 on the PFV surface. This interaction acts like a “molecular zipper,” bridging adjacent vesicle membranes. As siRNA progressively associates with the vesicle surfaces, contact regions expand, generating membrane strain and curvature stress. To relieve this strain, the vesicles undergo membrane fusion, rupture, and reorganization, forming multilamellar structures that trap siRNA within the reorganized lipid bilayers. This dynamic vesicle-to-multilayer transformation reflects a form of energy minimization driven by the neutralization of electrostatic charges. The process bypasses the need for ethanol or any organic solvent to initiate self-assembly, relying purely on ionic and mechanical forces at the nanoscale. Importantly, the efficiency of this solvent-free encapsulation is comparable to that of traditional ethanol-based microfluidic mixing while eliminating the requirement for solvent removal or post-processing. Further, in efforts towards green chemistry, sustainable alternative approaches for LNP-mRNA encapsulation, Tang et al. demonstrated the use of soya bean oil for LNP encapsulation with mRNA or siRNA nucleic acid targets [89]. Taken together, Figure 2 not only maps the diversity of existing LNP synthesis strategies but also highlights a clear evolutionary trend, from solvent-dependent, microfluidics-based systems toward greener, solvent-free innovations. This shift reflects a broader rethinking of how lipid self-assembly, encapsulation, and scalability can be achieved with fewer environmental and operational constraints. Figure 6 provides model data and outcomes from pioneering works in this non-conventional encapsulation works.

## 4. Process Conditions for Non-Conventional LNP Encapsulation Approaches

While conventional LNP production methods, such as ethanol injection and microfluidic mixing, have been widely adopted, their scalability and flexibility remain constrained by process parameters, including solvent choice, shear stress, and mixing precision (Table 1). To overcome these limitations, several non-conventional encapsulation strategies have emerged, leveraging advances in process intensification and nanomanufacturing. These approaches include ultrasonic cavitation, high-pressure homogenization, micro-mixing via membranes, and solvent-free self-assembly. Each offers unique control over droplet size, particle uniformity, and encapsulation efficiency, enabling improved formulation performance across diverse payload types such as mRNA, siRNA, and small molecules (Table 1).

**Table 1:** Process parameters and key details on the various non-conventional LNP encapsulation approaches. Direct numerical comparisons across studies in Table 1 should be interpreted with caution, as the reported metrics are not standardized and vary with experimental conditions (e.g., formulation composition, mixing parameters, and analytical methods) and payload type; accordingly, these values are intended to provide an indicative overview rather than a harmonized benchmarking of methods.

Method	Process Conditions	mRNA/Payload Info	Analytics	Reference
Preformed Vesicles	Three-inlet microfluidic droplet junction system with SM-102:DSPC:Chol: DMG-PEG2000 (50:10:38.5:1.5 mol%). TFR: 2 mL/min, FRR: 3. Post-encapsulation by microfluidics or manual pipetting into preformed vesicle solutions.	FLuc mRNA (1922 nt), EGFP mRNA (997 nt).	Physicochemical characteristics and in vitro expression; long-term storage stability tested.	[47,48]
Microfluidic Ultrasonication Cavitation (MUC) Both lab scale and large scale.	Custom ultrasonic microreactor with 20 mM citrate buffer. Lipid mix: 50:10:38.5:1.5 molar ratio. N/P ratio: 6; various flow rate ratios. Flow Rates: Lipid–ethanol and aqueous streams (1–10 mL/min) merged via microchannels. Ultrasound Frequency: ~20–40 kHz, applied downstream of mixing. Temperature: Maintained at ~25–30 °C to prevent mRNA denaturation.	T7 polymerase transcribed mRNA, 4486 nt.	DLS (size, PDI), Qubit RNA Assay (EE), cryo-TEM, and in vivo transfection efficiency.	[87]
Hot Homogenization	Temperature: 5–20 °C above lipid melting point. Pressure: 800–1000 bar. Rapid cooling post-emulsion.	Applied to small molecules and mRNA; typical sizes range from 50 to 120 nm.	DLS (size, PDI), zeta potential, TEM, and EE assessed via HPLC or fluorimetry.	[68,69,70]
Hot Melt Extrusion (HME) with High-Pressure Homogenization	Extrusion temperature: 60–90 °C. Screw speed optimized. Homogenizer pressure: 500–1000 bar. Cooling: Immediate post-emulsion cooling to solidify LNPs.	Applied for mRNA and dual payloads (e.g., miRNA + drug). Particle size ~ 94 nm, EE ~ 93%.	DLS (size, PDI), zeta potential, EE, pharmacokinetics, biodistribution.	[71,72]
SMART-MaGIC Extrusion	Syringe-based extrusion using a 21G nozzle at 200 kPa. DSPC:Chol:DOTAP: DMG-PEG (10:48:40:2) in ethanol. N/P ratios varied from 0.1 to 125—dialysis with a 3.5 kDa membrane.	LNP-mRNA at 1 µg/mL. Lipid:mRNA ratio 2:1. Size control via N/P optimization.	DLS, zeta potential, and encapsulation screening at varied N/P. Cryo-TEM, storage stability.	[55]
Membrane Micromixing	SPG ceramic membrane (2–10 µm pores). Flow rates: 500–1000 mL/min. Veth/Vaq: 1/4 or 1/6. Post-treatment with filtration and dialysis.	DOTAP-based mRNA LNPs. Size ~ 103 nm, PDI ~ 0.15, EE > 95%, Yield: 85–56%.	DLS, cryo-TEM, EE (fluorimetry), storage study (2 months).	[75]
Thin Film Hydration (One-pot synthesis)	Lipids dissolved in organic solvents were evaporated to form a thin film and rehydrated with aqueous mRNA, followed by sonication or extrusion. All lipid components and payloads combined in a single vessel—no organic solvent—using mild heating (~37–45 °C) to induce spontaneous nanoparticle formation.	Typical for hydrophobic and hydrophilic payloads, with post-hydration mRNA encapsulation.	DLS, EE (RiboGreen), zeta potential, cryo-TEM.	[72,74]
Self-Assembly (Solvent-Free)	Mild heating (37–45 °C), mixing lipid and mRNA in aqueous buffer. No organic solvents. Thermodynamically driven NP formation.	Applicable to ionizable lipids and naked mRNA. EE typically >90%.	DLS, fluorescence assay, stability monitoring, gel electrophoresis.	[69,70,88]
Emulsification Approaches	Double emulsion (W/O/W) under 40–60 °C. Stabilizers added to prevent coalescence—organic phase removal via evaporation or diffusion.	Used for mRNA and peptide payloads. Can achieve a small size of ~100 nm.	DLS, UV-Vis, gel electrophoresis, PDI and EE quantification.	[70,72]

## 5. Comparison of Conventional vs. Non-Conventional LNP Production Approaches

Although conventional LNP formulation methods remain the industry standard due to their simplicity and established regulatory familiarity, non-conventional production methods are rapidly gaining attention for their potential to improve scalability, process control, and product quality. Conventional approaches—such as manual pipette mixing, vortexing, or microfluidic mixing—are straightforward and reproducible; however, they often face challenges in terms of batch-to-batch consistency, throughput, and energy efficiency. In contrast, non-conventional strategies integrate process intensification concepts (e.g., continuous-flow synthesis, solvent-free emulsification, or high-pressure homogenization), offering greater control over nanoparticle formation and improved adaptability to industrial-scale production.

Table 2 presents a comparative overview of conventional and non-conventional LNP production methods. It outlines the respective advantages and disadvantages of each technique, including factors such as reproducibility, scalability, encapsulation efficiency, solvent requirements, and thermal sensitivity. This comparison highlights the increasing demand for hybrid or modular systems that integrate the robustness of conventional mixing with the precision and sustainability of modern intensified processes.

## 6. LNP Manufacturing and Large-Scale Production Challenges

Conventional LNP production methods, such as microfluidic encapsulation, rely on high volumes of organic solvents, specialized mixing equipment, and labour-intensive purification steps [24]. These processes can be expensive due to low solvent recovery rates and stringent regulatory requirements. In contrast, emerging methods, such as solvent-free microfluidic devices with continuous-flow systems, show promise for reducing costs by improving yield efficiency, minimizing solvent waste, and simplifying downstream processing. These approaches employ modular encapsulation and the construction of functional LNPs with mRNA complexes for therapeutic and vaccine applications. While initial capital investment for new technology might be high, the long-term operational costs per dose can be significantly lower compared to conventional solvent-based methods. However, these cost estimates are a result of modelling and would require robust field studies and surveys to compare the impacts. Table 3 and Table 4 compare the small-scale and large-scale LNP production aspects and the different emerging and well-established techniques. While Table 3 compares the different approaches and the commercially available tools and instrumentation, Table 4 compares the feasibility of small-scale and large-scale LNP encapsulation approaches.

One of the primary challenges in LNP manufacturing has been translating laboratory-scale protocols into industrial production. Traditional methods often struggle with reproducibility and scalability, limiting their economic feasibility for the mass production of therapeutics, such as mRNA vaccines. Key scalability limitations include maintaining consistent batch quality, preserving the biological activity of encapsulated molecules, the need for extensive screening, and batch-to-batch variability [2,3]. Hence, it is crucial to highlight current trends in scalable LNP production [40].

Presently, most biopharmaceutical manufacturing relies on batch processes [24]. Each production step requires pre- and post-process testing, making the workflow labour-intensive and time-consuming, while still prone to significant batch variability. Consequently, the demand for trained personnel is high, and, along with reagent costs, this accounts for nearly 80% of total manufacturing expenses for mRNA technology [1,3]. A more efficient alternative should therefore minimize repetitive testing steps, comply with FDA regulations, and align with quality-by-design (QbD) principles [1,7,96]. Ideally, such an approach would support both small-scale and large-scale production within the same facility, thus reducing time and resource expenditure. Small-scale production for clinical and preclinical trials typically requires around 1000 doses, whereas commercial-scale production can involve up to 10 million doses over a few days [6,8,40]. Consequently, a single facility must be capable of operating across production volumes differing by up to four orders of magnitude—a major logistical and technological challenge [1]. To address this, continuous biomanufacturing (CBP) has emerged as a promising solution. Ouranidis et al. emphasize the importance of developing continuous rather than batch manufacturing techniques, as continuous methods enable greater uniformity, automation, and reproducibility. Integrating CBP with in-line quality monitoring and real-time process analytical technology (PAT) can facilitate faster proof-of-concept validation at the laboratory scale and ensure consistent scalability to commercial production [97]. The US Food and Drug Administration (FDA) defines continuous manufacturing processes as processes involving continuous “feeding of input materials”, “transformation of in-process materials”, and “removal of output materials” [97,98,99].

Many LNP manufacturing methods already fit this definition. Microfluidic mixing (Table 4), for example, is set up with continuous flow. Therefore, LNPs’ raw materials enter and exit the manufacturing setup without interruption. Lipids and mRNA flow into the microfluidic channels, lipids aggregate and encapsulate mRNA within the mixers of the microfluidic channels, and then LNPs are extracted from the microfluidic channels. As a counterexample, conventional pipette-based mixing operates as a batch process: a single batch of lipids and mRNA is mixed to generate a single batch of LNPs, and each additional production run requires preparing and mixing a new batch of these components [100]. This discontinuous flow of raw materials increases the PDI of manufactured LNPs. Regulations require LNPs’ PDI to be below 0.30 [99]; therefore, continuous manufacturing processes facilitate easier compliance with regulations.

Another critical aspect of LNP manufacturing within a CBP framework is selecting the synthesis technique. Traditional large-scale preparation methods still face substantial limitations, primarily due to technological needs [101]. Khairnar et al. (2022) describe high-pressure homogenization (HPH) as a promising scale-up approach that has been successfully applied to LNP production [102]. Industrial HPH units, such as the LAB 60 homogenization system, can process up to 1.2 tons of material per hour and can be operated in parallel to increase throughput. This technique also reduces contamination risks and has demonstrated success at both medium (40–50 mL volume batches) and large scale, with operating rates of up to 12 L/h production scales [5]. However, its high operating pressure—ranging from 3.5 to 207 MPa—poses a significant drawback. Excessive pressure can damage encapsulated biomolecules, limiting their applicability for sensitive formulations [103]. Furthermore, variable shear forces within the system lead to particle polydispersity, complicating regulatory approval [4].

Microfluidic mixing remains the current gold standard for GMP-compliant LNP manufacturing because it offers well-controlled, reproducible nanoparticle formation under defined mixing regimes, enabling robust batch-to-batch consistency in critical quality attributes (e.g., size, PDI, encapsulation, and potency). Importantly, microfluidics has substantial regulatory familiarity, with established precedents across clinical and commercial programmes and mature quality-by-design (QbD) frameworks linking critical process parameters to product performance. The approach also integrates readily with validated in-line/at-line analytics and process analytical technology (PAT) strategies for real-time monitoring and control, facilitating reliable scale-up/scale-out and streamlined deviation management. These advantages—together with standardized quality-control workflows and documented manufacturing robustness—continue to support microfluidics as a benchmark platform against which emerging non-conventional routes should be compared. Molecular simulations have shown that continuous production using staggered Y-shape herringbone mixers could yield mRNA-LNP vaccines at scales sufficient for hundreds of millions of doses, with projected minimum prices ranging from €1.25 to €1.55 per dose [104]. While microfluidics enables fine control and scalability, it still faces limitations, including residual solvent content, channel clogging, complex device fabrication, and the need for extensive parameter optimization [13].

Overall, these observations highlight the need for ongoing improvement of LNP biomanufacturing technologies. Although microfluidics currently represents the most promising pathway toward standardized, scalable production, its limitations suggest that hybrid strategies may be necessary. For instance, emulsification or ultrasound-based methods can enable solvent-free LNP production but suffer from batch variability, as shown in Table 2. Integrating such methods with microfluidic systems could combine the advantages of both, providing solvent-free operation alongside the reproducibility and process control required for clinical and industrial applications.

**Table 3:** Overview of the LNP formulation techniques and scalability between lab-scale and commercial-scale. Direct numerical comparisons across studies in Table 3 should be interpreted with caution, as the reported metrics are not standardized and vary with experimental conditions (e.g., formulation composition, mixing parameters, and analytical methods) and payload type; accordingly, these values are intended to provide an indicative overview rather than a harmonized benchmarking of methods.

	Lab Scale				Large Scale			
Approach	Typical Volume Range	Volume Scale-Up	Equipment	Ref	Typical Volume Range	Volume Scale-Up	Equipment	Ref
High-speed homogenizers	Shear-based mixing for pre-emulsification (e.g., W/O and W/O/W). Adjustable speed and rotor-stator heads are used for pre-emulsion.	1–500 mL	Ultra-Turrax T25 (IKA)	[73]	Reduce droplet size, create uniform nanoemulsions. Requires high pressure (1000–2000 bar) and is scalable.	Litres to 100+ L	Avestin EmulsiFlex-C5	[71]
Probe sonicators	Ultrasonic energy to reduce droplet size. Risk of heating or RNA degradation unless cooling is applied	0.5–200 mL	Branson SFX250 Sonification unit	[105]	Mean particle size between 120 and 400 nm	Well-established	NA	[106]
Vortex mixers/Overhead stirrers	Low-energy emulsification (pre-mix). Often used with surfactant-stabilized systems	5–250 mL	Heidolph Hei-TORQUE Value 100	[62]	No data found			
Microfluidic chips	Continuous small-volume generation of monodisperse droplets. Flow-focusing or T-junction designs for high control	10–1000 µL/min	NanoAssemblr Benchtop (Precision NanoSystems)	[26]	Continuous-flow synthesis with high control. Scalable via number-up strategy	Up to 10 L/h (parallel arrays)	NanoAssemblr GMP System	[6]
Mini-extruders or membrane emulsification	Membrane emulsification with nanoscale pores. Used for size refinement (e.g., 100 nm)	~100–500 µL per cycle	Avanti Mini-Extruder (Avanti Polar Lipids)	[107]	Controlled droplet generation through porous membranes. Low energy input; scale-up is challenging but promising Crossflow Membrane System (Micropore Technologies)	10–100 L	BioX, Cellink, USA	[55]
Syringe pump systems	Flow control in microfluidic or membrane setups. Essential for reproducible flow-driven emulsions.	Variable	Harvard Apparatus PHD Ultra	[22,25,26,108]	No data found			
Hot melt extrusion + homogenization	No data found				Single-step scalable solvent-free nanoparticle production. Best for thermostable payloads; integration with downstream	10–100 L	Leistritz Micro-18 Extruder	[72]
One-pot or Thin film hydration	siRNA entrapment requires thermal cycling and brief sonication for self-assembly	200–500 µL	Branson ultrasonic cleaner, 2510	[55]	Limited to large-scale.			
Staggered Herringbone mixer	Over 270 million doses, 200 L/min	Batch approach, up to 100 mL	Mostly R&D PDMS-based devices	[109]	Continuous, mean particle size from 20 to 200 nm. Over 270 million doses	200 L/min	PDMS-based microfluidic devices	[1,97,110]
Ethanol injection	No data found				Well-established	Mean particle size, 120–400 nm	n.a.	[106]

**Table 4:** Comparison of challenges associated with lab-scale and commercial-scale manufacturing of LNP-mRNA encapsulation by emerging non-conventional methods and feasibility for continuous manufacturing.

Approach	Lab-Scale Challenges	Commercial-Scale Challenges	Continuous Manufacturing Feasibility	References
Microfluidic Mixing	Precise control of flow rates; clogging in narrow channels; batch-to-batch variability due to manual prep	Maintaining laminar flow at high throughput, equipment fouling, and difficulty scaling parallel devices	High potential; commercial units like NanoAssemblr Scale-Up platform already demonstrate CM feasibility	[111]
Membrane Micromixing	Reproducibility issues with membrane pore uniformity; high shear may degrade mRNA	Membrane fouling; scale-up limited by pressure requirements and membrane durability	Moderate potential with modular scaling; CM integration requires membrane regeneration protocols	[62,74,75,112]
Thin-Film Hydration	Requires solvent evaporation; batch-dependent rehydration quality; large PDI.	Low reproducibility at scale; challenging to automate; slow rehydration kinetics	Low CM feasibility due to batch nature; recent automation trials are promising but limited	[88,113]
Ethanol Injection	Rapid dilution is necessary to prevent aggregation; however, it is inefficient at small scales for high concentrations.	Handling of large volumes of organic solvents; solvent recovery systems are needed	Moderate CM potential; commercial ethanol injection setups exist but require validation for GMP	[92,114]
Self-Assembly (Bulk)	Low encapsulation efficiency; sensitive to ionic strength and temperature; hard to control size	Poor control of mixing conditions; scale-up requires stringent SOPs and in-line monitoring	Low to moderate; CM possible with continuous feeding, though not standard yet	[73]
Emulsification	Surfactant selection impacts RNA stability; droplet size heterogeneity	Phase separation issues; poor reproducibility in stirred tank reactors	Moderate CM feasibility; continuous stirred-tank reactors with in-line droplet size control tested	[62]
Hybrid Polymer–Lipid NPs	Complexity in component assembly; difficult to achieve uniform particles	Polymer scalability issues; reproducibility across batches is challenging	Emerging CM strategies under development; need modular plug-and-play lipid/polymer input systems	[62,115]
Lyophilization + On-site Assembly	Freeze–thaw damage; poor reconstitution fidelity without cryoprotectants	Equipment cost, moisture content control, and validating shelf-stability	Promising in modular point-of-care platforms; not yet standard for CM	[47,70,116]
High-Pressure Homogenization	Particles tend to be polydisperse, and biomolecules may get damaged	High energy is needed, and production units can get damaged over time	High potential; Widespread and easy to use devices, can be installed in sequence to avoid damage; continuous LNP production has been shown before	[68,102,117]

## 7. Economic Perspectives of Different Emerging and Conventional mRNA-LNP Encapsulation Approaches

The lipid nanoparticle (LNP) manufacturing market is currently estimated to be worth USD 0.38 billion (2024). It is projected to expand to USD 2.53 billion by 2035, driven by a compound annual growth rate (CAGR) in the range of ~11–22% [101,118]. This large projected growth underscores the extraordinary demonstration of the significance of LNP production as an enabling technology for nucleic acid therapeutics (especially mRNA). Key cost drivers in LNP manufacturing include capital investment in specialized mixing, microfluidic, and purification infrastructure; consumables (e.g., single-use fluidic assemblies, chromatography media, and membranes); lipid raw materials (ionizable lipids, PEG-lipids, and helper lipids); and process yield losses associated with scale-up, residual solvent removal, and quality control. The techno-economic analysis of upstream (in vitro transcription (IVT) of the mRNA), downstream (purification and stabilization of the mRNA), and formulation by encapsulations and preservation using excipients associated cost analysis was compared in detail with cost-per-dose comparison across different vaccine platforms in [24,104,119,120].

A useful benchmark in the published works on the techno-economic modelling of mRNA vaccine production is provided by Kis et al., which evaluated the overall resource and time requirements to meet pandemic-scale demands. They found that the drug substance (RNA) and drug product (LNP encapsulated) stages impose high operational expenditures (OPEX), especially due to raw materials and downstream purification steps (e.g., tangential flow filtration and chromatography) [104]. In a related cost model, the drug product cost per dose for mRNA vaccines was estimated at approximately USD 1.05 (interquartile range: 0.81–1.35), compared to approximately USD 0.38 for adenoviral vector vaccines and USD 0.40 for self-amplifying RNA (saRNA) platforms [120]. Thus, LNP-formulated mRNA currently carries a cost premium relative to viral and self-amplifying RNA modalities.

When contextualizing within the broader vaccine production scope, comparative studies (e.g., techno-economic analysis of Novavax’s protein subunit platform, “BICS”) suggest that mRNA platform costs per dose can be ~7× higher than baculovirus insect cell line systems and saRNA counterparts, largely driven by high raw material costs such as CleanCap reagents, nucleotides, and lipid excipients [119,121]. Meanwhile, fill-finish and facility overheads remain significant fixed-cost burdens in both LNP and conventional vaccine platforms. Additionally, LNP-mRNA vaccines may necessitate a cold-storage chain, leading to increased logistics and distribution costs per unit vaccine/dose.

For emerging non-conventional LNP formulation methods (e.g., solvent-free, PFVs, and membrane micromixing), techno-economic implications become critical. On the upside, reducing dependence on organic solvents can lower costs associated with solvent handling, removal, and safety. Simplifying mixing (e.g., via self-assembly or macromixing and one-pot synthesis methods) may also reduce capital requirements for microfluidics or homogenization modules. However, trade-offs exist: alternative methods often yield lower results, lead to increased lipid excess (to facilitate encapsulation), or encounter scaling challenges that increase the cost per dose. The literature on LNP encapsulation highlights that excess lipid is often used to enhance encapsulation; however, this approach eases cost and toxicity, particularly when scaled [122]. Additionally, the need to evaporate large volumes of solvent (or to flush residual solvents) in large batches can be time-intensive and cost-prohibitive in conventional solvent-based processes. To date, most non-conventional approaches are in the proof-of-concept, technology readiness level (TRL) 3/4 stages of development and will require extensive clinical validation and analytical characterization, followed by cell-line-based in vitro and animal model-based in vivo studies, before being compared with conventional methods of LNP-mRNA encapsulation processes [122].

From the market perspective, the strong projected growth in LNP manufacturing indicates that service providers and Chief Marketing Officers (CMOs) will play a key role in absorbing capital burdens and enabling access to specialized infrastructure. Indeed, the Roots Analysis report, cited ultimate ties in this work, emphasizes that many sponsors outsource LNP manufacturing to specialist providers to leverage cost efficiencies, expertise, and flexibility. During the LNP encapsulations, Moderna outsourced its encapsulation process to Precision Nanosystems, primarily for efficient encapsulation and downstream applications. Another way to reduce the cost per dose is by having a dual payload or multiple payload encapsulated to ensure dual or multifunctional mRNA-LNP delivery systems, analogous to polyvalent protein or toxin subunit vaccines. This is another key criterion that many leaders in the mRNA manufacturing space actively explore.

In summary, while LNP-based mRNA therapeutics currently incur higher per-dose costs compared to more mature vaccine modalities, emerging non-conventional formulation techniques offer opportunities to reduce capital and operational expenditures, provided that yield, robustness, and scalability are adequately optimized. The upcoming decade is likely to see increased competitive pressure and innovation in process intensification, material cost reduction, and modular manufacturing architectures, making LNP-based therapeutics economically viable for broad clinical deployment.

## 8. Research Directions and Future Outlook on Emerging Methods of LNP Formulation

### 8.1. Innovations in Emerging Methods of LNP Encapsulations and Formulation Strategies, DOE and Digital Twins

One of the key research priorities in mRNA–LNP technology today is the stabilization of LNPs at room temperature through solid, lyophilized formulations. This advancement is critical for expanding the global reach of mRNA therapeutics and vaccines, particularly in low-resource settings where cold-chain infrastructure is limited. A significant driver of the high cost of mRNA–LNP-based therapeutics is the loss of efficacy due to cold-chain failures and the lack of dose flexibility, as many formulations are restricted to multi-dose vials without on-demand modularity. Consequently, there is increasing interest in emerging encapsulation strategies such as prefabricated, freeze-dried LNPs that can be later rehydrated with mRNA, enabling rapid, point-of-care modular assembly [17,47].

In this context, lyophilization (freeze-drying) must be understood not only as a storage innovation but as an integral component of formulation workflows. For example, non-conventional synthesis methods—including membrane micromixing (membrane contactor), thin-film hydration (one-pot), and emulsification approaches—are increasingly being adapted to generate LNPs that are compatible with solid-state stabilization. One study demonstrated that a one-pot alcohol dilution–lyophilization technique allowed efficient mRNA loading while eliminating the need for solvent removal, resulting in thermostable gene expression-capable formulations [123].

Such techniques often allow greater control over particle morphology, lipid–RNA interaction strength, and buffer compatibility—all critical for lyophilization success and reconstitution fidelity [116]. A Design of Experiments (DoE) approach was employed by Wang et al. (2024) to optimize lipid ratios and identify a lyophilized mRNA–LNP formulation that retained greater than 90% encapsulation and transfection efficiency post-reconstitution, reinforcing the importance of co-optimizing formulation and storage [70].

Furthermore, methods such as self-assembled polymer–lipid hybrids, nanoemulsions, and polymeric nanocapsules have shown promise in enabling single-dose, thermostable formulations, thereby reducing reliance on complex equipment or cold storage [14]. These strategies also align with ongoing techno-economic optimization efforts, where simplification of formulation pipelines (fewer unit operations, smaller equipment footprint) and enhancement of product shelf-life can substantially lower OPEX and expand deployment in diverse clinical and global health scenarios.

Ultimately, integrating lyophilization-readiness and digital twin simulations into the design criteria of next-generation LNP–mRNA formulations is not merely a downstream stability consideration—it is becoming a front-end design constraint. Digital twin platforms, incorporating multivariate machine learning and real-time process feedback, are being used to simulate encapsulation dynamics, drying kinetics, and CQAs during lyophilization to better guide development [124,125]. This emerging paradigm ensures that non-conventional synthesis approaches are engineered from the outset for robust, scalable, and cost-effective mRNA therapeutics.

### 8.2. AI-Driven Optimization in the Conventional and Emerging LNP Production Processes

Artificial intelligence (AI), particularly machine learning (ML), is transforming drug discovery and manufacturing by its ability to reduce total experiments for optimization, a rational predictive approach to select appropriate excipients, lipid combinations, buffer compositions, etc., especially for LNPs and emerging non-conventional carriers like nanoemulsions and polymeric systems. Maharjan et al. (2023) identified over 30 ML programmes used across the biopharma pipeline, with models like random forest (RF), support vector machines (SVMs), and deep neural networks (DNNs) predicting optimal excipient combinations to enhance LNP stability and mRNA encapsulation efficiency [115]. These models have enabled formulation optimization, predictive quality control, and in silico screening of LNP-based systems. For instance, Boost has been employed to predict mRNA stability across different LNP compositions [126], while CNNs have been used for high-throughput imaging analysis of nanoparticle morphology [125,127]. ML thus facilitates rapid, cost-effective design of excipient combinations and scale-up simulation in non-conventional formulations like emulsions or PLGA-encapsulated mRNA systems [61,62]. Castillo-Hair et al. (2021) further demonstrated how supervised learning can optimize mRNA untranslated regions to enhance translation and immunogenicity [125]. ML-driven in silico design has replaced traditional LNP formulation screening. For instance, Ding et al. (2023) screened over 300 lipid candidates to predict encapsulation performance [124], thereby reducing lab time and improving reproducibility. In parallel, clustering algorithms have classified diverse nanoformulations and revealed hidden structure–function links in alternative systems, such as polymeric nanospheres and emulsions [128]. In upstream biomanufacturing, ML models are applied to predict optimal IVT reaction conditions and purification strategies, integrating multi-omic datasets for better control of critical quality attributes (CQAs) such as particle size and polydispersity [127]. This is especially critical for LNPs made via microfluidic, thin-film hydration, or emulsion techniques, where model-guided selection of lipids and surfactants can significantly impact performance and safety. Thus, future mRNA–LNP platforms are increasingly integrating molecular dynamics (MD) simulations and AI-driven lipid libraries to co-optimize mRNA sequences and nanoparticle parameters, advancing non-conventional methods such as microfluidic self-assembly, hybrid polymer–lipid systems, and lyophilized powders for thermostable delivery [115,125]. Bayesian and Gaussian process-based DoE frameworks now enable precision tuning of lipid ratios (ionizable:helper:cholesterol:PEG) and encapsulation efficiency in microfluidic settings. At the same time, ML models have screened >300 proprietary lipids for saRNA and circular RNA delivery, identifying candidates with improved endosomal escape and reduced immunogenicity [124].

### 8.3. Coencapsulation of mRNA and Relevance of Personalized Medicine Toward LNP Synthesis

Multi-payload delivery using lipid nanoparticles (LNPs) has emerged as a key strategy to enhance the breadth of vaccines and therapeutic versatility. Moderna’s bivalent mRNA vaccine, mRNA-1273.214, combines mRNAs encoding both the ancestral and Omicron variant spike proteins in a 1:1 ratio, each at 25 µg, using a single LNP formulation (co-delivery approach). This co-encapsulation enables the simultaneous translation of two distinct antigens from a single dose, thereby enhancing immunogenicity across variants while maintaining a manageable particle size and polydispersity [129,130]. Notably, LNPs have a favourable capacity to encapsulate multiple mRNA strands up to ~15–20 kb combined, which distinguishes them from siRNA carriers that face size constraints of less than ~1 kb [2]. The self-amplifying mRNA (saRNA) approach offers additional advantages, as it encodes replicase machinery that amplifies the RNA transcript intracellularly, allowing antigen expression from ultra-low doses (e.g., 0.1–10 µg per dose) compared to conventional mRNA vaccines (30–100 µg). saRNA systems thus reduce the required LNP payload, allowing more room for co-formulated adjuvants or RNA combinations, and enhancing thermostability due to the lower total RNA concentration [131,132,133]. Emerging non-conventional LNP synthesis methods, such as microfluidic co-flow encapsulation, electrostatic self-assembly, and lyophilized prefabricated LNPs, are enabling flexible and modular systems for on-demand payload loading, particularly beneficial for decentralized manufacturing. For instance, microfluidic mixing enables precise control of FRR and TFR, maintaining particle size distributions below 100 nm with a PDI of less than 0.2, even when co-encapsulating multiple mRNAs or saRNA strands [24]. Prefabricated, lyophilized LNP cores can be stored at room temperature for over 12 months with minimal degradation (<10% mRNA loss), and rehydrated with custom payloads on-site, as demonstrated in pandemic-response pilot studies [134]. Such platforms reduce dependency on cold-chain logistics by over 60% and eliminate the need for high-volume extrusion equipment [132,133]. Additionally, co-encapsulation efficiency for two mRNAs in dual-payload LNPs exceeds 80% when using ethanol–aqueous phase ratios optimized between 3:1 and 4:1. These advancements are paving the way for thermostable, scalable, and dose-adaptable vaccines, offering a robust route for multi-payload RNA therapeutics, including bivalent, trivalent, or even multiplexed saRNA vaccines in future outbreak preparedness scenarios [2,131].

### 8.4. Process Cost Modelling and Techno-Economic Modelling

Process cost modelling is essential for evaluating the feasibility of emerging mRNA–LNP platforms, particularly those diverging from traditional microfluidics-based systems. While conventional LNP manufacturing has been rigorously assessed in terms of capital (CAPEX), OPEX, and cold-chain logistics [104], non-conventional strategies such as self-assembled lipid–polymer hybrids, point-of-care microfluidic encapsulation, and lyophilized formulations—remain underexplored.

Prefabricated LNP libraries, for instance, eliminate the need for centralized large-scale microfluidics but introduce cost drivers in on-site reconstitution, dose modularity, and container-device integration [115]. Integrating lyophilization into early design criteria incurs additional costs, including increased cycle time (by ~20–30%), buffer selection constraints, and monitoring of the glass transition temperature [70]. These variables must be incorporated into cost models using unit operation breakdowns and stochastic parameters such as batch failure probabilities or RNA loss during freeze–thaw cycles.

Quantitative modelling using Bayesian DoE and digital twins is now feasible. For example, Monte Carlo simulations have been used to explore RNA–lipid interaction landscapes, while Gaussian process regression predicts optimal ratios of ionizable, helper, cholesterol, and PEG. These in silico strategies help minimize experimental waste and can predict OPEX reductions of up to 25–40% when identifying scalable, low-shear encapsulation methods. However, dry powder LNPs, inhalable formulations, and nanocapsule-based vaccines still lack published process cost estimates. Their potential to reduce cold-chain reliance and enable single-dose thermostable vaccines could significantly lower global deployment costs—but only if models account for equipment footprint, stability data, and supply chain complexity.

### 8.5. GMP Compliance and Regulatory Considerations for LNP-Based Therapeutics

While conventional large-scale processes rely on well-characterized, batch-based unit operations with fixed process analytical technologies (PATs), emerging strategies demand flexible GMP models that accommodate decentralized, continuous, or on-demand production [6,135]. For instance, microfluidics-based LNP assembly enables high control over particle size and homogeneity. Still, it introduces difficulties in in-line sterility validation, multi-fluid contact surface validation, and continuous process monitoring, all of which are essential for GMP adherence [26,27]. Furthermore, platform technology approaches for LNPs must include detailed documentation of lipid composition variability, encapsulation efficiency, and RNA integrity across scales [7]. Non-conventional approaches, such as pre-formed LNP cores or dry-powder reconstitution, require stability studies and modular fill–finish workflows under GMP-compliant environments [106,107]. Continuous manufacturing holds promise, but regulatory requirements are still evolving, as existing GMP frameworks favour batch genealogy over fluid dynamic production models [12,40,96]. As such, aligning emerging encapsulation modalities with evolving regulatory science, including Pharma 4.0 and QbD principles, will be critical for accelerating the scalable and compliant deployment of mRNA–LNP therapeutics [97,136].

## 9. Conclusions

This review examined the limitations of conventional mRNA–LNP manufacturing. It highlighted a suite of emerging, non-conventional encapsulation strategies designed to overcome key barriers in scalability, stability, cost, and adaptability. Techniques such as membrane micromixing, hot homogenization, SMART-MaGIC extrusion, water-in-oil emulsification, and self-assembly were explored for their potential to enable solvent-free, modular, and thermostable formulations. These methods offer enhanced encapsulation flexibility, including multi-payload and lyophilized LNP formats, and align with green chemistry and point-of-care manufacturing paradigms. The integration of digital twins, Bayesian DoE, and AI-driven lipid library screening further advances the predictive formulation landscape, allowing for rational optimization of lipid ratios, RNA compatibility, and cost modelling. Process cost modelling remains a frontier area for non-conventional systems, but early data suggest potential OPEX reductions of up to 40%, with improved stability and shelf-life metrics. In summary, membrane-based mixing/contactor approaches appear among the closest non-conventional options to complement or partially replace microfluidics for clinical-grade mRNA-LNP production, largely due to their short processing times, scalable throughput, and compatibility with continuous or semi-continuous operation. Coacervation/phase-separation strategies are likewise promising for translation because they can enable highly efficient nucleic-acid complexation/compaction and may be engineered toward large-scale, solvent-minimized workflows. However, robust control of critical quality attributes will be essential. In contrast, prefabricated (modular) vesicle/post-insertion platforms are particularly well-suited for early-stage formulation screening and resource-limited settings, enabling flexible “mix-and-match” assembly and rapid evaluation across payloads (including shorter RNAs such as guide RNAs), while additional validation is typically required to demonstrate GMP robustness at scale. Their success, however, hinges on addressing regulatory, analytical, and scale-up challenges. When coupled with precision biomanufacturing tools, these innovations pave the way toward more cost-effective, thermostable, and globally deployable RNA-based treatments.

## Figures and Tables

**Table 2:** Advantages and disadvantages of conventional and non-conventional LNP production approaches.

	Method	Pros	Cons	References
Conventional Method	Microfluidic mixing	High reproducibility, controlled particle size, scalable with proper setups	Requires organic solvent; high mRNA loss due to dead volume; costly microfluidic chips	[88,95]
	Manual pipette mixing or vortexing	Simple, no special equipment needed	Poor reproducibility; batch-to-batch variability; not scalable	[12]
Non-Conventional Method	Thin film hydration	Easy to implement, suitable for lipid film preparation	Low encapsulation efficiency; requires solvents; risk of mRNA degradation	[2]
	Hot homogenization	Organic solvent-free, scalable	High temperature can degrade lipids and mRNA payloads	[70]
	Cold homogenization	Organic solvent-free, scalable, Better for temperature-sensitive compounds	Limited encapsulation efficiency; requires multiple passes for size reduction.	[88]
	Water/oil/water emulsion	Potential for high encapsulation efficiency	Complex process; solvent residue risk; stability concerns	[95]
	Emulsification/ ultrasound/ sonication	Solvent-free possible; small size achievable	Shear stress may damage mRNA; batch variability	[12,88]
	Emulsification–solvent diffusion	Good for lipophilic drug inclusion	Uses organic solvents; risk of mRNA damage	[9]
	Emulsification–solvent evaporation	Well-established for hydrophobic drugs	Organic solvent required; multi-step process; not ideal for RNA	[2]
	Membrane contactor	Continuous, scalable, good size control	Expensive setup; requires fine-tuning; solvent use possible	[47,48]

## Data Availability

No new data were created or analyzed in this study.
